# Using Mini-Grants to Build Multi-Sector Partnerships in Rural Tennessee

**DOI:** 10.13023/jah.0102.08

**Published:** 2019-07-06

**Authors:** Ginny Kidwell, Kristine Bowers, Taylor M. Dula, Randolph F. Wykoff

**Affiliations:** East Tennessee State University College of Public Health; East Tennessee State University College of Public Health; East Tennessee State University College of Public Health; East Tennessee State University College of Public Health

**Keywords:** Appalachia, health care, rural health care, socioeconomic status, health outcomes, economics

## Abstract

Rural counties in Tennessee, including those located in Appalachia, face some of the greatest health challenges in the nation. Unpublished data collated by the East Tennessee State University College of Public Health (ETSU) show that Tennessee’s 52 Appalachian counties vary dramatically from its 43 non-Appalachian counties in virtually all socioeconomic, behavioral, and health outcome metrics. Since 2011, the Tennessee Institute of Public Health (TNIPH) has actively encouraged local communities to address behavior change, enhance educational achievement, and improve economic conditions as essential components for improving health and well-being in rural Tennessee.

Rural counties in Tennessee, including those located in Appalachia, face some of the greatest health challenges in the nation. Unpublished data collated by the East Tennessee State University College of Public Health (ETSU) show that Tennessee’s 52 Appalachian counties vary dramatically from its 43 non-Appalachian counties in virtually all socioeconomic, behavioral, and health outcome metrics. Since 2011, the Tennessee Institute of Public Health (TNIPH) has actively encouraged local communities to address behavior change, enhance educational achievement, and improve economic conditions as essential components for improving health and well-being in rural Tennessee (Figure 1).

To encourage and assist local communities in developing programs in these components, TNIPH created and implemented the Regional Roadmaps series of “mini-grant” programs across rural Tennessee. The purpose of these programs was to support the development of community-level, multi-sectoral partnerships that link health, economic development, and education as catalysts to improving health and well-being in those communities. A mantra evolved from these projects: “Everyone benefits from a healthy, educated, drug-free workforce.” This mantra was effective at speaking to, and facilitating the involvement of, a broad cross-section of community members. It helped them understand the essential inter-dependence of multi-sectoral efforts in improving health and well-being. This understanding was key to their willingness to work across sectors to identify and address their community’s health challenges.

The mini-grants were implemented in four separate projects:

1. Regional Roadmap for a Healthier Appalachian Tennessee (November 2013–March 2015) in the Appalachian counties of Tennessee;
2. Healthy WEST: **W**orking to **E**nergize and **S**trengthen **T**ennessee (June 2015–June 2016) in rural West Tennessee counties outside of Memphis;
3. Regional Roadmap 2: Down the Road to a Healthier Tennessee (April 2016–March 2017) in the Appalachian counties of Tennessee; and
4. Healthy MID TN: **M**eaningful **I**mprovements **D**esigned **T**hrough **N**etworks (October 2017–December 2018) in Middle Tennessee counties outside the Appalachian region and Nashville.

The mini-grant program in the Appalachian region of the state was funded primarily by the Appalachian Regional Commission (ARC) and BlueCross BlueShield of Tennessee Health Foundation with additional funds from the Niswonger Foundation and Eastman Foundation. Funding for counties outside Appalachia came primarily from the BlueCross BlueShield of Tennessee Health Foundation. Additional support was provided by the ETSU College of Public Health, University of Wisconsin Population Health Institute, and a variety of regional partners.

To qualify for a mini-grant, a local community had to create a working partnership with representatives from economic development, health, and education sectors with additional partners encouraged. These partnerships then had to identify their own health priorities and what actions they were willing to take to address these priorities prior to applying for a mini-grant from TNIPH. To support these partnerships, TNIPH held mandatory pre-application sessions that highlighted best practices, reviewed relevant data, and offered technical assistance in reporting and program evaluation.

Over the first 5 years of this project, a total of $217,500 was awarded as $2500 (75 grants) and $5000 (6 grants) mini-grants to community-based organizations representing 81 programs in 87 of the 95 Tennessee counties. Fifty-one (63%) programs were awarded to communities within the 52 Appalachian counties of Tennessee. While each partnership was required to have representatives from all three sectors, the identified primary applicant was from the health sector in 18%, from business and industry in 20%, education in 12%, nongovernmental organizations in 41%, and local government in 10% (Table 1). The projects were highly variable but generally fell into the categories of physical activity, community education, clinical, nutrition, substance abuse, and others.

This experience shows that mini-grants can be a highly effective and relatively inexpensive mechanism to build regional partnerships, encourage inter-sectoral collaboration, and empower communities to identify and address their health challenges. An important lesson from this project is that one of the most effective ways to engage community partners is to reach out initially to local economic development entities as primary points of entry. Another important lesson is that the organizing agency, in this case TNIPH, benefited from having a strong network of partners across the state. The statewide TNIPH project network included numerous partners and contacts throughout Tennessee that reflects years of community engagement. The members of this network can both participate in, and promote, the mini-grant process.

Finally, the experience with the mini-grant process reinforced the importance of allowing local autonomy in identifying regional health priorities. Local control and direction put many of the variables in the hands of those who had the most to gain from the success of their efforts. It is important to recognize that the metrics of success for a rural mini-grant project are not necessarily the traditional “outcome” metrics, usually reported for public health projects (e.g., weight loss for an obesity project or knowledge gained for an education program). Rather, the metric of success should be seen as the ability of a community to either create a multi-sectoral partnership that did not previously exist or to reinforce an already existing framework that, through collaborative effort, was able to identify and address a local health priority. By producing benefits that reach beyond the project scope, the Regional Roadmaps approach becomes a model for replication in rural communities across the nation.

## Figures and Tables

**Figure 1:**
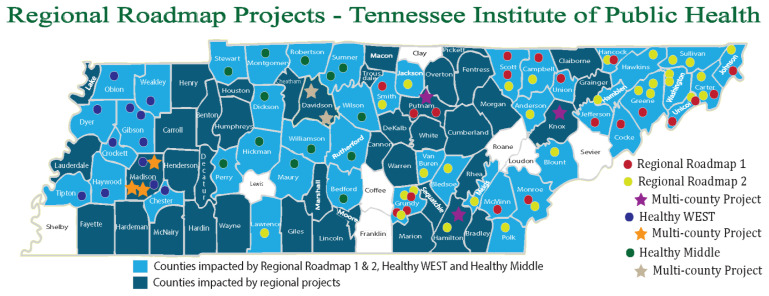
Regional Roadmap Projects Map

**Table 1:** Appalachian Regional Roadmaps Lead Organizations

Lead Organizations	# of Organizations	%
Health (H)	9	18
Business and industry (BI)	10	20
Education (ED)	6	12
Non-governmental organizations (NGO)	10	41
City/County governments (CC)	8	10
**Total**	**51**	**100**

